# Mitochondrial ROS driven by NOX4 upregulation promotes hepatocellular carcinoma cell survival after incomplete radiofrequency ablation by inducing of mitophagy via Nrf2/PINK1

**DOI:** 10.1186/s12967-023-04067-w

**Published:** 2023-03-25

**Authors:** Chao Peng, Xi Li, Feng Ao, Ting Li, Jingpei Guo, Junfeng Liu, Xiaoting Zhang, Jinyan Gu, Junjie Mao, Bin Zhou

**Affiliations:** 1 Department of Interventional Medicine, The Fifth Affiliated Hospital of Sun Yat-sen University, Zhuhai, 519000 Guangdong China; 2 Guangdong Provincial Key Laboratory of Biomedical Imaging and Guangdong Provincial Engineering Research Center of Molecular Imaging, The Fifth Affiliated Hospital of Sun Yat-sen University, Zhuhai, 519000 Guangdong China; 3 Department of Anesthesiology, Gansu Provincial People’s Hospital, Lanzhou, 730000 Gansu China; 4 Library Department, The Fifth Affiliated Hospital of Sun Yat-sen University, Zhuhai, 519000 Guangdong China

**Keywords:** Mitochondria, Mitophagy, HCC, NOX4, Nrf2, ROS

## Abstract

**Background:**

The recurrence of hepatocellular carcinoma (HCC) after radiofrequency ablation (RFA) remains a major clinical problem. Cells that survive the sublethal heat stress that is induced by incomplete RFA are the main source of HCC relapse. Heat stress has long been reported to increase intracellular reactive oxygen species (ROS) generation. Although ROS can induce apoptosis, a pro-survival effect of ROS has also been demonstrated. However, the role of ROS in HCC cells exposed to sublethal heat stress remains unclear.

**Methods:**

HepG2 and HuH7 cells were used for this experiment. Insufficient RFA was performed in cells and in a xenograft model. ROS and antioxidant levels were measured. Apoptosis was analyed by Annexin-V/PI staining and flow cytometry. Protein expression was measured using western blotting. Colocalization of lysosomes and mitochondria was analyzed to assess mitophagy. Corresponding activators or inhibitors were applied to verify the function of specific objectives.

**Results:**

Here,we showed that sublethal heat stress induced a ROS burst, which caused acute oxidative stress. This ROS burst was generated by mitochondria, and it was initiated by upregulated NOX4 expression in the mitochondria. n-acetylcysteine (NAC) decreased HCC cell survival under sublethal heat stress conditions in vivo and in vitro. NOX4 triggers the production of mitochondrial ROS (mtROS), and NOX4 inhibitors or siNOX4 also decreased HCC cell survival under sublethal heat stress conditions in vitro. Increased mtROS trigger PINK1-dependent mitophagy to eliminate the mitochondria that are damaged by sublethal heat stress and to protect cells from apoptosis. Nrf2 expression was elevated in response to this ROS burst and mediated the ROS burst-induced increase in PINK1 expression after sublethal heat stress.

**Conclusion:**

These data confirmed that the ROS burst that occurs after iRFA exerted a pro-survival effect. NOX4 increased the generation of ROS by mitochondria. This short-term ROS burst induced PINK1-dependent mitophagy to eliminate damaged mitochondria by increasing Nrf2 expression.

## Introduction

Radiofrequency thermal ablation (RFA) is widely considered to be an effective local therapy for hepatocellular carcinoma (HCC) because of its minimal invasiveness and limited complications [1]. However, although the outcomes after RFA appear to be comparable to those reported after surgical resection when the tumor diameter is < 1 cm, HCC recurrence after RFA remains a major problem and is associated with a poor prognosis [2]. For patients with HCC tumors less than 3 cm in diameter, five-year recurrence rates are reported to reach 50–70% [3, 4]. Destroying all the malignant cells within the target region is the primary aim of RFA treatment. However, tumor heterogeneity, the quality of imaging guidance, variation in the achieved temperature within a tumor, and the heat sink effect are factors that lead to incomplete RFA (iRFA) [1]. Cancer cells in the iRFA zone are exposed to sublethal hyperthermia, and they consequently either undergo apoptosis or recover from reversible injury [5]. The survival of cells in the sublethal hyperthermia zone is the main cause of HCC recurrence after RFA. Thus, targeting the molecular mechanisms that facilitate HCC cell survival after iRFA may increase the ablation zone and improve prognosis.

Hyperthermal treatment has been reported to increase intracellular reactive oxygen species (ROS) levels [6–9]. Modulation of intracellular ROS levels is crucial for cellular homeostasis, as cells respond differently to varying levels of ROS [10]. Toxic levels of ROS can kill tumor cells, while the nonlethal increase in ROS levels facilitates tumor progression and metastasis [10]. Sustained exposure to heat stress (> 1 h) can induce sufficient ROS accumulation and eventually lead to cell death [6, 7, 11, 12]. However, HCC cells in the iRFA zone suffer a state of hyperthermia for approximately 15 min at temperatures between 38 and 50 °C [1]. Most cells that are exposed to such heat stress do not die and instead exhibit greater migration and invasion abilities [13–15], which indicates that the increased ROS levels under such stress conditions may be nonlethal. At nonlethal levels, ROS can act as signaling molecules to activate stress-responsive pathways [10, 16]. Mildly elevated ROS levels can activate the PI3K-Akt pathway, and ERK1/2 pathway, upregulate heat shock protein expression (HSP) [17–19], and induce autophagy [16], which have all been reported to be crucial for survival during heat stress. Moreover, ROS has been reported to promote the survival of liver endothelial cells suffering from ischemia/reperfusion injury [20], the adaptation and survival of isolated hepatocytes [17] and the survival of human placenta-derived multipotent cells [21]. Thus, we postulate that the changes in ROS levels may also contribute to the survival of HCC cells after iRFA. However, the exact dynamic change in ROS levels and the related mechanism have not yet been fully explored.

Mitophagy is a type of selective autophagy that mediates the clearance of damaged mitochondria and is involved in survival [22]. ROS have been reported to induce PINK1-dependent mitophagy [23–25]. Short-term sublethal heat exposure can induce mitochondrial depolarization [25] and partially damage the outer mitochondrial membrane of isolated mitochondria [26]. However, the mitochondrial electron transport chain (ETC), which is the source of mitochondrial ROS (mtROS) located at the inner mitochondrial membrane, is not markedly affected by short sublethal heat exposure [26]. The generation of more mitochondrial ROS under sublethal heat stress conditions may require a trigger. Recently, “ROS-induced ROS release” was reported to be a mechanism underlying ROS augmentation [27]. In addition to mitochondria, NADPH oxidases are another main source of ROS in mammalian cells [16]. There are seven isoforms of NADPH oxidase (NOX1–5 and DUOX1–2) [16]. It has been reported that NADPH oxidases (NOX2 and NOX4) can localize to the mitochondria and affect mtROS generation [28–30]. NOX4 has also been reported to be upregulated by heat stress [31]. Moreover, ROS generated by NADPH oxidases have been shown to inhibit necrosis or apoptosis in many types of cells [31–36]. However, little is known about the role of NADPH oxidases in ROS production in the context of iRFA, and mitophagy has not been investigated as a mechanism that can be initiated by ROS and is involved in survival.

In this study, we show that the upregulation of NOX4 in mitochondria increases mtROS generation, which triggers PINK1-dependent mitophagy to clear mitochondria that are damaged by sublethal heat stress by increasing the expression of Nrf2, thereby promoting cell survival after iRFA. This pro-survival effect of ROS may be an important mechanism underlying the heat stress resistance of HCC cells induced by iRFA.

## Materials and methods

### Reagents

N-acetylcysteine (NAC), trypan blue and MitoTEMPO were obtained from Sigma‒Aldrich (USA). Diphenyleneiodonium chloride (DPI), VAS2870, Mdivi-1, carbonyl cyanide 4-(trifluoromethoxy) phenylhydrazon(FCCP), lucigenin, bardoxolone and 2',7'-dichlorodihydrofluorescein diacetate (DCFH-DA) were obtained from MCE(USA), and NADPH was purchased from Beyotime Biotechnology (China). MitoTracker™ Deep Red, MitotrackerFM Green and MitoSOX™ were obtained from Invitrogen (UK). Specific antibodies were purchased from different companies as follows: GAPDH, LC3B,Nrf2 (CST, USA), NOX4, P62, TOMM20, VDAC1, Cleaved caspase-3 (Abcam, UK), BCL-2, and BAX (Affinity, China). The mitochondrial stress test complete assay kit (ab232857) and extracellular oxygen consumption assay kit (ab197243) were obtained from Abcam(UK).

### Sublethal heat treatment

An in vitro model of insufficient thermal ablation was established based on the method described in a previous report, with minor modifications [37]. Briefly, HCC cells were seeded in 6-well plates. The cells reached 70–80% confluence after 24 h of culture. Then, medium that was prewarmed at 46 °C was added to the wells. The cells were subsequently submerged into a water bath that was preheated at 46 °C for 15 min. After with the medium was replaced with fresh medium that had been kept at 37 °C, the cells were returned to the incubator. NAC (5 mM) or MitoTEMPO (500 nM) was administered 1 h after heat treatment and incubated for 4 h. DPI (10 μM) and VAS2870 (10 μM) were added 30 min after heat treatment and incubated for 4 h. FCCP (1 μM), bardoxolone(0. 5 μM) and Mdivi-1 (10 μM) were used for pretreatment for 1 h.

### Cell viability assays

Cells were seeded and reached 70–80% confluence after 24 h of culture. The cell viability was first determined before heat treatment. The cells were then exposed to 46 °C for 15 min, and cell viability was measured at 0, 2, 6, 12, and 24 h. At each indicated time point after heat treatment, the cells were trypsinized and harvested by centrifugation (1200 × rpm, 4 min). The cells were resuspended and mixed with 0.4% trypan blue solution. Cell viability was evaluated by counting the number of cells that excluded the dye.

### ROS and mitochondrial superoxide production measurement

ROS production was measured using DCFH-DA. MitoSOX™ Red was used to measure mitochondrial superoxide production. Both indicators were dissolved in dimethyl sulfoxide (DMSO) and diluted to working concentrations in Hank’s balanced salt solution. Cells exposed to heat stress were trypsinized and harvested at the indicated times. Then, the collected cells were resuspended in PBS with either 10 μM DCFH-DA or 5 mM MitoSOX™ Red and incubated at 37 °C for 20 min. The fluorescence signal was measured by using flow cytometry(CytoFLEX) at 485 nm/538 nm for DCFH-DA and 510 nm/590 nm for MitoSOX™ Red.

### Determination of the glutathione (GSH) levels

A commercial glutathione assay kit(Beyotime, China) was used for GSH level measurement. The experiment was carried out based on the manufacturer’s instructions. Cells were collected by centrifugation(1000 × rpm,5 min). The precipitates were mixed with a triple volume of protein removal reagent. After being frozen twice, the cells were centrifuged at 10000 × g for 10 min and the supernatants were collected. The GSH detection agent was mixed and 150 μL was added per well. After adding 10 μL of sample and incubating at room temperature for 5 min, 50 μL of NADPH was added, and the absorbance of the well was measured at 412 nm. The GSH levels were calculated based on a standard curve.

### Measurement of total antioxidant capacity (T-COA) and superoxide dismutase (SOD) activity

The total antioxidant capacity assay was conducted based on the manufacturer’s instructions (Beyotime, China). Cells were washed and transferred to a homogenizer. The homogenates were centrifuged at 12000 × g for 5 min. The total antioxidant capacity detection agent was prepared based on the instructions, and 200 μL was added per well. After adding 20 μL of sample and incubating, the absorbance of the wells was measured at 734 nm. A commercial total SOD activity assay kit (Beyotime, China)based on WST-8 was used. The cells were lysed and centrifuged at 12000 × g for 5 min. The supernatants were collected. The working solution was prepared based on the instructions. Twenty microliters of sample was mixed with SOD detection buffer and WST-8 and incubated at 37 °C for 30 min. The absorbance of the well was measured at 450 nm. The T-COA and SOD activities were calculated based on a standard curve.

### Measurement of NADPH oxidase activity

The NADPH oxidase activity of total cell homogenate was assessed by superoxide production. The method was based on lucigenin chemiluminescence described previously [36, 38]. Briefly, cells were lysed in lysis buffer (Beyotime, China) and the proteins were harvested by centrifugation(10000 × rpm,15 min).Working buffer(phosphate buffer(50 mM),EDTA(1 mM),sucrose(150 mM) was prepared. After the protein concentrations were measured, 50 μg of protein was added to 500 μL of working buffer. Then, dark-adapted lucigenin(100 μM) was added. Chemiluminescence was measured immediately after NADPH (100 μM) was added at 15 s intervals for 1 min by an EnVision® luminometer.

### Mitochondrial membrane potential (MMP) measurement

TMRE was used to assess the MMP. Cells exposed to heat stress or not were treated with NAC or DMSO for the indicated times.Then, the cells were incubated with medium containing 150 nM TMRE. After staining for 20 min at 37 °C, cells were washed, and the fluorescence signal was acquired by flow cytometry (CytoFLEX).

### Determination of apoptosis

An Annexin V/PI apoptosis detection kit (BD Biosciences) was used to assess apoptosis. Briefly, cells were trypsinized without EDTA and harvested by centrifugation(200 × g, 5 min). Then, 100 μL of 1 × binding buffer was used to resuspend the cells. Five microliters of Annexin V and PI were added. After incubation for 15 min at room temperature in the dark, the samples were immediately assessed by using a flow cytometer (CytoFLEX). Apoptotic cells were divided into cells undergoing early(Annexin V + /PI −) or late apoptosis (Annexin V + /PI +). A total of 10,000 cells per condition were analyzed for these measurements..

### Small interfering RNA (siRNA) -mediated knockdown

Human NOX4 siRNA was obtained from GenePharma (China). The siRNAs were transfected into cells by using Lipofectamine® 3000 Reagent (Life Technologies) based on the manufacturer’s protocol. Twenty-five picomoles of siRNA per well was used for transfection. The sequences of siRNAs was NOX4 siRNA(5′- AACCUCUUCUUUGUCUUCUACAUGCUGCU − 3′) and control siRNA(5′-UUCUCCGAACGUGUCACGUTT -3′).

### RNA isolation and real-time PCR

An RNeasy kit (QIAGEN, USA) was adopted to extract total RNA from cultured cells. PrimeScript™ RT Master Mix (Takara, Japan) was used for genomic DNA digestion and reverse transcription. Real-time PCR was performed using SYBR® Green Master Mix (Applied Biosystems) on a thermal cycler (Bio-Rad, USA). GAPDH was chosen as an internal control. The primer sequences of the genes were described as follows:NOX1-F 5'-GGTCAACACGAGGAGAGC-3ʹ; NOX1-R: 5'-CAAGGATCCACTTCCAAGACTC-3'; NOX2-F: 5'-CCCAATCCCTCAGTTTGCT-3'; NOX2-R: 5'-CCTTCTGTTGAGATCGCCAA-3'; NOX3-F: 5'-ACCTTCTGTAGAGACCGCTAT-3'; NOX3-R:5'-TCACATGCATACAAGACCACA.

-3'; NOX4-F:5'-CTGTGGTGTTACTATCTGTATTTTCTC-3''; NOX4-R:5'-CTTGCTGCATTCAGTTCAACA-3'; NOX5-F: 5'-GCCAGTGCCTCAACTTCG-3';

NOX5-R:5'-CCACTACCACGTAGCCCATA-3'; DUOX1-F: 5'-GCGTCTACATG

AGAAATGCCA-3'; DUOX1-R: 5'-GCAGCAGTGCATCCACAT-3'’; DUOX2-F:5'-CGCCACCTACCAGAACATC-3''; DUOX2-R: 5'-GGTAGAGAAGAACTGCTC

AGAG-3'. RIPK1-F: 5′-TCCTCGTTGACCGTGAC-3′; RIPK1-R: 5 -GCCTC

CCTCTGCTTGTT-3′; RIPK3-F: 5′-CCAGCTCGTGCTCCTTGACT-3′:; RIPK3-R: 5′-TTGCGGTCCTTGTAGGTTTG-3′; GAPDH-F:5'-TGACAACAGCCTCAAGAT-3'; GAPDH-R:5'-GAGTCCTTCCACGATACC-3'.

### Western blotting analysis

Cells were collected and lysed using RIPA lysis buffer (Beyotime, China) containing 1% protease inhibitor on ice. The supernatants containing proteins were harvested by centrifugation(10,000 × rpm,15 min) at 4 °C. Equal protein extracts were separated using SDS‒PAGE (15% or 10%) and then transferred onto polyvinylidene difluoride membranes. After blocked with 5% BSA solution, the membranes were incubated with the relevant antibodies at 4 °C overnight.

### Separation of nucleari and mitochondrial fractions

A commercial kit(Beyotime, China) was used to separate separating mitochondria. Based on the manufacturer’s protocol, cells were trypsinized and harvested by centrifugation(200 × g,10 min). After washing with PBS, 1 ml mitochondrial isolation solution was added for 10 min on ice, and then the cells were transferred into a homogenizer. Then, the homogenates were centrifuged at 600 × g for 10 min at 4 °C. The supernatants were collected and centrifuged at 11000 × g for 10 min at 4 °C. The precipitates contained mitochondria and were harvested. The nuclear protein extraction reagent was added to the precipitates..After vortexing, nuclear proteins were harvested by centrifugation(16000 × g, 10 min). The supernatants were collected.

### Mitochondrial DNA (mtDNA) extraction and copy number quantification

Mitochondria were lysed with mitochondrial lysis buffer on ice for 10 min. Then, 5 μL of enzyme mix was added. After incubation in a 50 °C water bath for 60 min, absolute ethanol was added and incubated for an additional 10 min at − 20 °C. mtDNA was collected by centrifugation at top speed for 5 min.The mtDNA was analyzed by PCR using MT-CO2 (mitochondrially encoded cytochrome c oxidase II): F: 5'-CAAACCTACGCCAAAATCCA-3', R:5'-GAAATGAATGAGCCTACAGA-3'. 18S rDNA (F:5'-TAGAGGGACAAGTGGCGTTC-3' and R:5'-CGCTGAGCCAGTCAGTGT-3') was choosed for measuring nuclear DNA.

### Immunofluorescence confocal microscopy (colocalization)

The cells were incubated with MitoTracker™ Deep Red (500 nM) for 10 min. After the medium was removed, the cells were washed, and 4% paraformaldehyde was added to fix for 15 min. Then, the cells were permeabilized using 0.2% Triton X-100 for 10 min. After a 30 min block with 5% BSA, primary antibody was added to the culture dish and incubated at room temperature for 30 min. FITC-conjugated secondary antibodies were added, and further incubated for 30 min. The cells were then stained with DAPI for 10 min. After washing, the cells were observed using a confocal microscope (Zeiss, Germany). MitoTracker™ Deep Red fluorescence was determined at wavelengths of 647/668 nm. All fields were imaged using a 63 × objective lens.

### Assessment of lysosome and mitochondria colocalization

Mitophagy was assessed by the colocalization of the mitochondria and lysosomes using a confocal system. The cells were incubated with MitoTracker™ Red (500 nM) for 10 min before heat treatment in an incubator. The cells were then washed with fresh medium and subjected to heat treatment. After 12 h, the cells were incubated with LysoTracker™ Green (50 nM) and Hoechst 33,342 for 15 min. The cells were then washed and observed with a Leica confocal system (Zeiss, Germany). MitoTracker™ Red fluorescence was measured at wavelengths of 647/668 nm and LysoTracker™ Green fluorescence was measured at wavelengths of 488/560 nm. Images were taken with a 63 × objective.

### Measurement of ATP levels

A commercial ATP determination kit(Beyotime, China) was used to measure intracellular ATP levels. After adding lysis buffer to the plates, the cells were scraped. The supernatants were harvested by centrifugation (12,000 × g, 5 min). Diluted ATP detection reagent was prepared on ice, and 100 μL was added per well. Then,20 μL of supernatant was then added per well. Luminescence was measured using an EnVision® illuminometer at 5 s after mixing. A BCA protein assay kit was used to measure theprotein concentrations of the supernatant. The ATP levels were normalized to the protein concentration of each sample.

### Oxygen consumption rate (OCR) measurement

Extracellular Oxygen Consumption Assay (Abcam, ab197243) and Mitochondrial Stress Test Complete Assay(Abcam, ab232857) kits were combined to measure the extracellular OCR according to the protocol. Briefly, approximately 4 × 10^4^ cells were seeded in a 96-well clear bottom black plate. After being cultured for the indicated times, the cells were subjected to sublethal heat stress or not. Medium containing NAC or not was added to the cells at 1 h after heat exposure. Ten microliters of fresh medium, oligomycin (15 μM), FCCP (25 μM), or antimycin A (15 μM) was added to the indicated wells 12 h after heat exposure. Then, high sensitivity mineral oil warmed to 37 °C was applied. Signal intensity was immediately measured using the TR fluorescence intensity mode at 380/650 nm by an EnVision® reader.

### TUNEL staining

Apoptosis was analyzed in 5 μm thick frozen sections of xenografts that were collected one day after iRFA using TUNEL staining. The sections were dried at room temperature for 20 min and then submerged in 4% paraformaldehyde to fix for 30 min. After washing twice with PBS, 0. 2% Triton X-100 was added and incubated at room temperature for 15 min. The sections were washed twice with PBS again. Equilibration buffer(1X,100 μL) was added and incubated at room temperature for 30 min. Then, TdT enzyme solution was added and incubated at 37 °C for 60 min. The sections were then stained with DAPI for 15 min. Fluorescence was measured using a fluorescence microscope (Leica,Germany). Apoptotic cells displayed red fluorescence under excitation. Blue fluorescence of DAPI was used to quantify the nuclei. Fiji software (ImageJ, Bethesda, MD) was applied to analyze the acquired images. TUNEL-positive cells were normalized to the total number of nuclei in each image.

### *Establishment of a subcutaneous HCC xenograft model and thermal ablation model *in vivo

The HCC xenograft model was established in BALB/c nude mice (male, 4–6 weeks) obtained from the Guangdong Medical Laboratory Animal Center (Guangzhou, China). Isoflurane anesthesia was used for the anesthesia of the animals. Surgical procedures were conducted under aseptic conditions. After local disinfection,HepG2 cells (2 × 10^6^) were injected subcutaneously into the right axilla of mice using 1 ml syringe. The experiment was not carried out until the diameter of subcutaneous tumors reached approximately 10 mm. The mice were randomly divided into four groups, namely, the control, iRFA, heat, and heat + iRFA groups, with each group including five mice. The RFA electrode was inserted into the tumor and positioned at one-third of the length of the tumor under ultrasound guidance. For iRFA, the ablation power was 5 W for 1 min. Sham ablation was conducted without ablation power. To evaluate postoperative recurrence, the tumor dimensions were measured every 4 days. The volume of tumors was calculated based on the formula: V = (length × width^2^)/2. On Day 20 after ablation,the mice were sacrificed and the tumor volume and weight were measured and recorded. The mice in the NAC or NAC + iRFA group were injected with 100 mg/kg NAC for 5 days via the tail vein starting 2 h after iRFA.

### Statistical analysis

All the data are expressed as the mean ± standard deviation. Statistical analysis was performed on SPSS Statistics 25 (USA) using Student’s t test and one-way analysis of variance (ANOVA). p < 0.05 was considered statistically significant. All the figures associated with statistics were generated with GraphPad Prism (Version 8.0).

## Results

### *Cells undergo oxidative stress after sublethal heat stress *in vivo

**Fig. 1:**
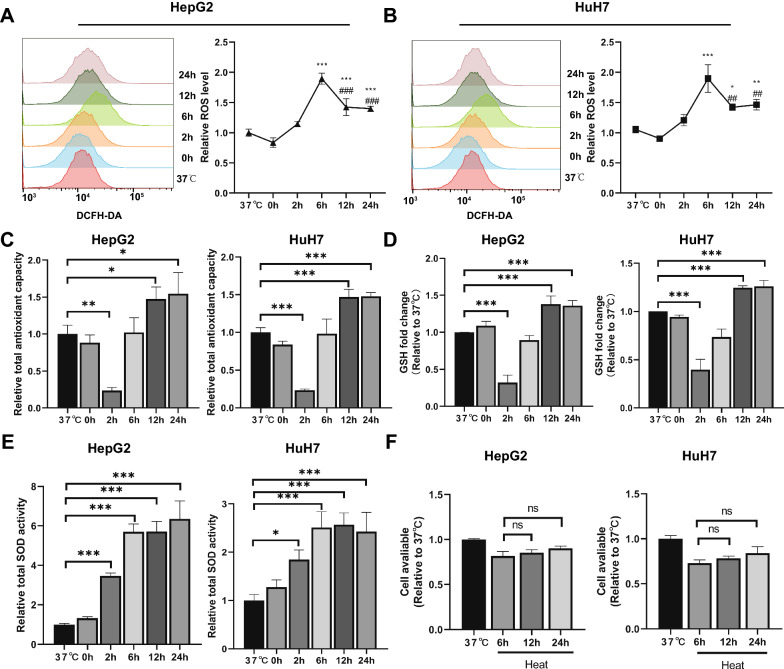
Dynamic changes in ROS production and antioxidant capacity after sublethal heat stress. HepG2 and HuH7 cells were incubated at 46 °C for 15 min or not. **A** The changes in ROS fluorescence **B** total antioxidant capacity, **C** reduced GSH level, and total **D** SOD activity were measured at the indicated time points (37 °C, 0, 2, 6, 12, and 24 h) after sublethal heat stress. **E** Cell viability measured at 37 °C,6, 12, and 24 h after sublethal heat stress. Values are the mean ± SE. *p < 0.05 vs. 37 °C; **p < 0.01 vs. 37 °C; ***p < 0.001 vs. 37 °C; ##p < 0.01 vs. 6 h; ###p < 0.001 vs. 6 h;*ns* no significance

To examine the dynamic changes in ROS levels after sublethal heat stress, we used a ROS indicator to determine the intracellular ROS levels at the indicated time points after sublethal heat stress. As shown in Fig. 1A, B, the ROS levels in both HepG2 and HuH7 cells gradually increased from 2 to 6 h after sublethal heat stress, then gradually decreased, and finally maintained at a level higher than that observed at 37 °C. As the antioxidant system is another factor that determines the fate of cells under oxidative stress, we also investigated the intracellular T-COA of cells at the indicated time points after sublethal heat stress. The results showed that it decreased in the first 2 h after sublethal heat stress, then gradually increased, and was finally maintained at a level higher than that observed at 37 °C (Fig. 1C). Therefore, we further separately quantified the levels of reduced GSH, a major antioxidant in cells, and total activity of SOD, an enzymatic antioxidant, after sublethal heat stress at the indicated time points. The results showed that the reduced level of GSH also gradually decreased from 2 to 6 h after sublethal heat stress, then gradually increased, and was finally maintained at a level higher than that observed at 37 °C (Fig. 1D). The total SOD activity gradually increased within 12 h after sublethal heat stress (Fig. 1E).These observations showed a transient impairment of antioxidant capacity and were in accordance with the dynamic changes in ROS levels after sublethal heat stress. Taken together, these data suggested that the cells undergo short-term oxidative stress after sublethal lethal heat stress. Given that oxidative stress is a trigger of cell death, we further assessed cell viability at 6, 12, and 24 h after sublethal heat stress. As shown in Fig. 1F, no significant cell death was observed in either HepG2 or HuH7 cells at 12 h or 24 h after sublethal heat stress, compared to 6 h after sublethal heat stress. This indicates that the ROS burst after sublethal heat stress was not strong enough to induce cell death.

### Inhibition of ROS production sensitizes cells to sublethal heat stress due to increased apoptosis

**Fig. 2:**
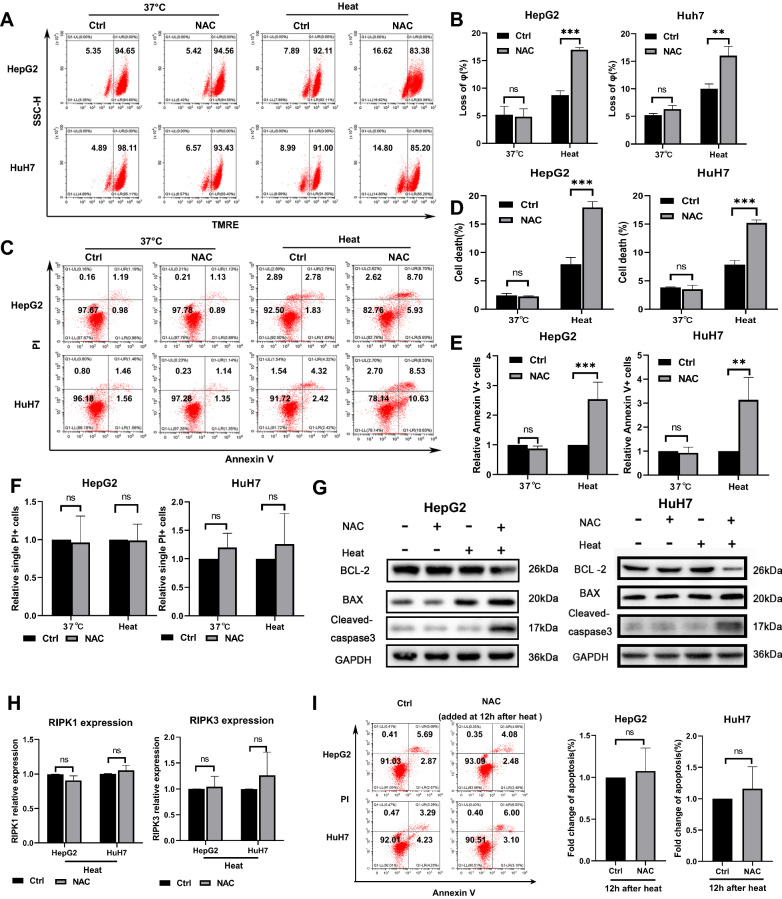
NAC induces apoptosis after sublethal heat stress. HepG2 and HuH7 cells that were untreated or subjected to sublethal heat stress were cultured for 12 h in medium with or without NAC (5 mM). **A** and **B** Mitochondrial membrane potential was assessed by TMRE staining and analyzed by flow cytometry at 12 h after sublethal heat stress. **C** and **D** Cell death was quantified using Annexin V-FITC/PI staining and flow cytometry at 24 h after sublethal heat stress. Summary graph representing the percentage of single PI + cells (Q1 areas) (**E**) and Annexin V + cells (Q2 and Q3 areas) (**F**) in each group. **G** Western blot of GAPDH, BAX, BCL-2, and Cleaved-caspase-3 expression. **H** mRNA expression of RIPK1 and RIPK3.HepG2 and HuH7 cells incubated with NAC(5 mM) at 12 h after sublethal heat stress. (I)Cell death was quantified using Annexin V-FITC/PI staining and flow cytometry at 24 h after sublethal heat stress. Values are the mean ± SE.**p < 0.01; ***p < 0.001; *ns* no significance

To further clarify the role of ROS after sublethal heat stress, we eliminated intercellular ROS by adding the ROS scavenger NAC 1 h after sublethal heat stress. First, we measured the MMP, a classical marker of the induction of cell death. Both HepG2 and HuH7 cells exhibited a significant decrease in the MMP at 12 h in the presence of NAC (Fig. 2A, B).This suggest that inhibition of ROS may cause cell death after sublethal heat stress. Then, cell death was assessed by flow cytometry using Annexin V-FITC/PI labeling. Cell death was significantly increased in the NAC treatment group (Fig. 2C, D). Notably, Annexin V + cells were the most significantly increased portion (Fig. 2C, E and F). This suggested that apoptosis is the main type of cell death induced by ROS inhibiton after sublethal heat stress. To further confirm the effect of ROS inhibiton, we measured the expression of apoptosis-related proteins. As shown in Fig. 2G, the protein levels of the active forms of Caspase-3 and BAX were increased, and the protein level of BCL-2 was decreased. In contrast, the mRNA levels of RIPK1 and RIPK3, markers of necroptosis, were not significantly altered (Fig. 2H). These results suggest that the ROS burst after sublethal heat stress exerts a pro-survival effect. The ROS burst after sublethal heat stress was confined to within 12 h (Fig. 1A). To further confirm the pro-survival effect of the ROS burst within 12 h after sublethal heat stress, we determined the effect on apoptosis by adding NAC 12 h after sublethal heat stress. As shown in Fig. 2I, no apoptosis was observed in the group treated with NAC at 12 h after sublethal heat stress. This suggests that the ROS burst within 12 h after sublethal heat stress contributed to the cells survival after sublethal heat stress.

### Enhanced production of mtROS driven by NADPH oxidase

**Fig. 3:**
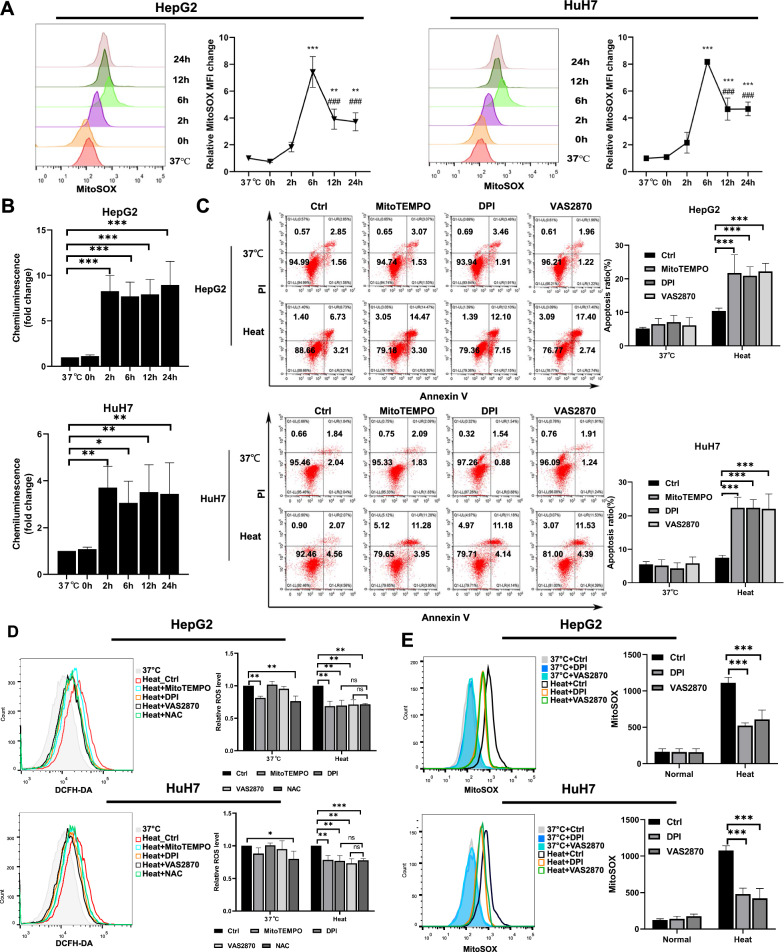
Elevated production of mitochondrial ROS driven by NADPH oxidases. **A** Changes in MitoSOX™ fluorescence as measured using flow cytometry at the indicated time points (37 °C, 0, 2, 6, 12, and 24 h) after sublethal heat stress.**B** Superoxide production by NADPH oxidase was measured in total cell homogenates. **C** Heated or unheated HCC cells incubated with or without inhibitors of ROS: NAC(5 mM), MitoTEMPO (500 nM), DPI (10 μM), or VAS2870 (10 μM). Apoptosis was quantified using Annexin V-FITC/PI staining and flow cytometry at 24 h after sublethal heat stress. **D** ROS levels as measured by flow cytometry at 6 h after sublethal heat stress. **E** Heated or unheated HCC cells preincubated with or without inhibitors of NADPH oxidase: DPI (10 μM) and VAS2870 (10 μM), MitoSOX™ fluorescence as measured using flow cytometry at 6 h after sublethal heat stress. Values are the mean ± SE.**p < 0.01 vs. 37℃; ***p < 0.001 vs. 37℃; ###p < 0.001 vs. 6 h; *ns* no significance

ROS can be generated in various cellular compartments, and they have different reactivities and stabilities. Mitochondria and cellular NADPH oxidase are the two main sources of cellular ROS [16]. To investigate the source of increased ROS production, mitochondrial superoxide production was assayed using MitoSOX™ staining, and NADPH oxidase activity was measured by the lucigenin-enhanced chemiluminescence method. As shown in Fig. 3A, mtROS levels gradually increased from 2 to 6 h after sublethal heat stress, then gradually decreased, and were finally maintained at a level higher than that observed at 37℃. NADPH oxidase activity also increased from 2 h after sublethal heat stress (Fig. 3B). Furthermore, both pharmacological inhibition of NADPH oxidase with DPI or VAS2870 and cleavage of mtROS with mitoTEMPO induced apoptosis and reduced the ROS levels after sublethal heat stress (Fig. 3C, D). This suggests that both sources contributed to the increased generation of ROS and cell survival after sublethal heat stress. Notably, the level of ROS reduction by NAC, a nonspecific ROS scavenger, was not more pronounced than that by mitoTEMPO, DPI, or VAS2870 (Fig. 3D). There may be an upstream and downstream relationship between mitochondria and NADPH oxidase. Reports suggest that NADPH oxidase is also located within mitochondria and induces mtROS release by generating ROS [29, 39, 40]. Therefore, we further investigated the effect of NADPH oxidase inhibition on mtROS generation. As shown in Fig. 3E, inhibition of NADPH oxidase by DPI or VAS2870 inhibited mtROS levels at 6 h after sublethal heat stress. Thus, NADPH oxidase is upstream of mtROS generation after sublethal heat stress.

### The upregulation of NOX4 in mitochondria contributes to the increase in ROS production after sublethal heat stress

**Fig. 4:**
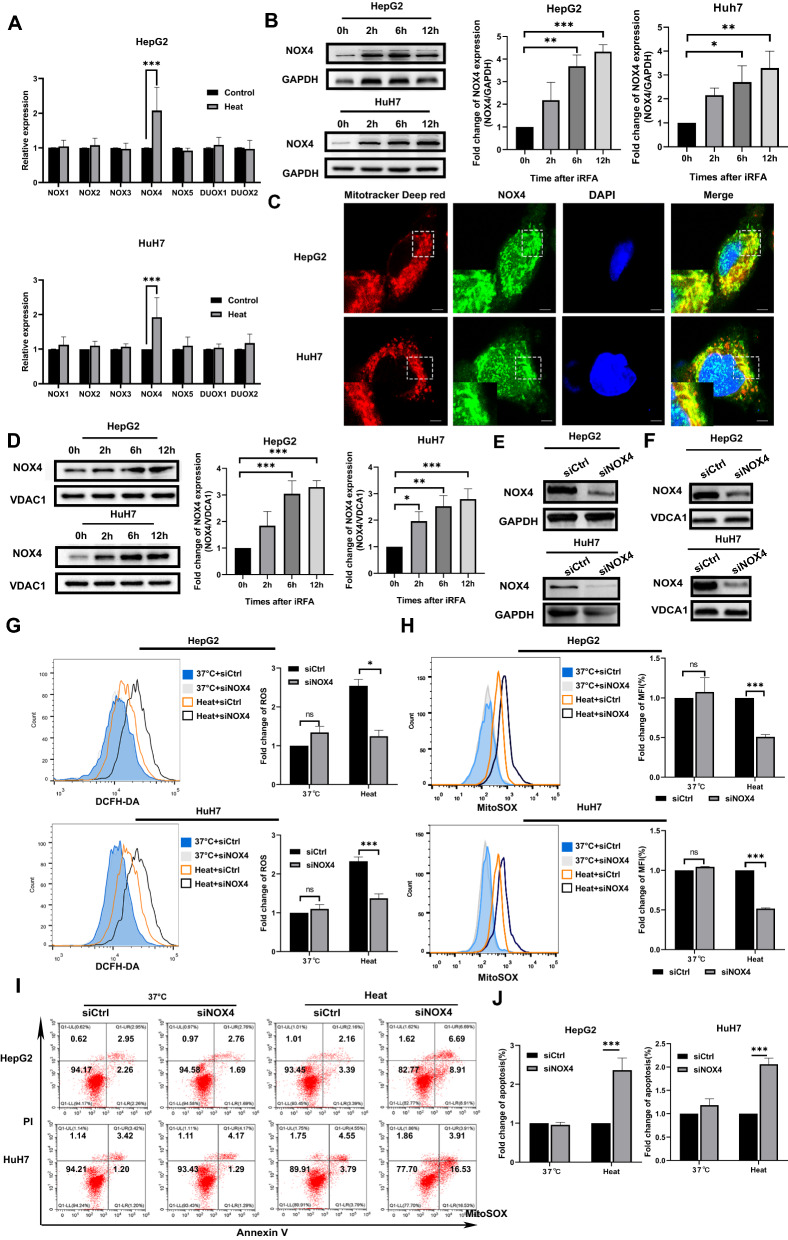
NOX4 localizes at mitochondria and contributes to the increase in ROS production after sublethal heat stress. **A** RT-qPCR analysis of NOX1–5 and DUOX1–2 mRNAs expression after sublethal heat stress. **B** Western blot of NOX4 expression in total cell lysates after sublethal heat stress. **C** Colocalization of mitochondria and NOX4. MitoTracker™ red and FITC- conjugated secondary antibody was used for NOX4. Nuclei were counterstained with Hoechst 33,342. Yellow displays colocalization. **D** Western blot of NOX4 expression in mitochondria after sublethal heat stress. **E** Protein levels of NOX4 in whole cell extracts. **F** Protein levels of NOX4 in mitochondria. **G** DCFH-DA and **H** MitoSOX™ fluorescence as measured using flow cytometry at 6 h after sublethal heat stress.**I** and **J** Apoptosis was quantified using Annexin V-FITC/PI staining and flow cytometry at 24 h after sublethal heat stress. Values are the mean ± SE. *p < 0.05;***p < 0.001; *ns* no significance

The NOX family contains seven members, and the mRNA expression of these genes was determined. The results showed that only NOX4 mRNA expression was significantly upregulated at 6 h after sublethal heat stress (Fig. 4A). Western blotting analysis also revealed that NOX4 protein expression was markedly increased in both cell types after sublethal heat stress (Fig. 4B). Studies have reported that NOX4 is located in mitochondria and is associated with the generation of mtROS in both breast cancer cells and renal cells [29, 41]. Therefore, we further investigated whether NOX4 is located in the mitochondria of HepG2 and HuH7 cells by using confocal microscopy. The overlap of NOX4 staining and MitoTracker™ red staining suggested that NOX4 localized at mitochondria in both HepG2 and HuH7 cells (Fig. 4C). To further verify this hypothesis, we measured NOX4 expression in the mitochondrial fractions of HepG2 and HuH7 cells by Western blotting. The results confirmed that NOX4 was located in the mitochondria, and its level was also increased in this compartment after sublethal heat stress (Fig. 4D). We then used siRNA-mediated knockdown of NOX4 to investigate its function (Fig. 4E). This approach was also successful in reducing mitochondrial NOX4 expression (Fig. 4F). Consistent with the pharmacological inhibition of NADPH oxidase, the knockdown of NOX4 significantly suppressed both total ROS and mtROS production at 6 h after sublethal heat stress (Fig. 4G, H). As expected, inhibition of NOX4 also increased apoptosis at 24 h after sublethal heat stress (Fig. 4I, J). These results suggest that upregulation of NOX4 in mitochondria is the trigger of increased mtROS generation after sublethal heat stress.

### Inhibition of ROS levels after sublethal heat stress induces the accumulation of damaged mitochondria and results in severe mitochondrial dysfunction

**Fig. 5:**
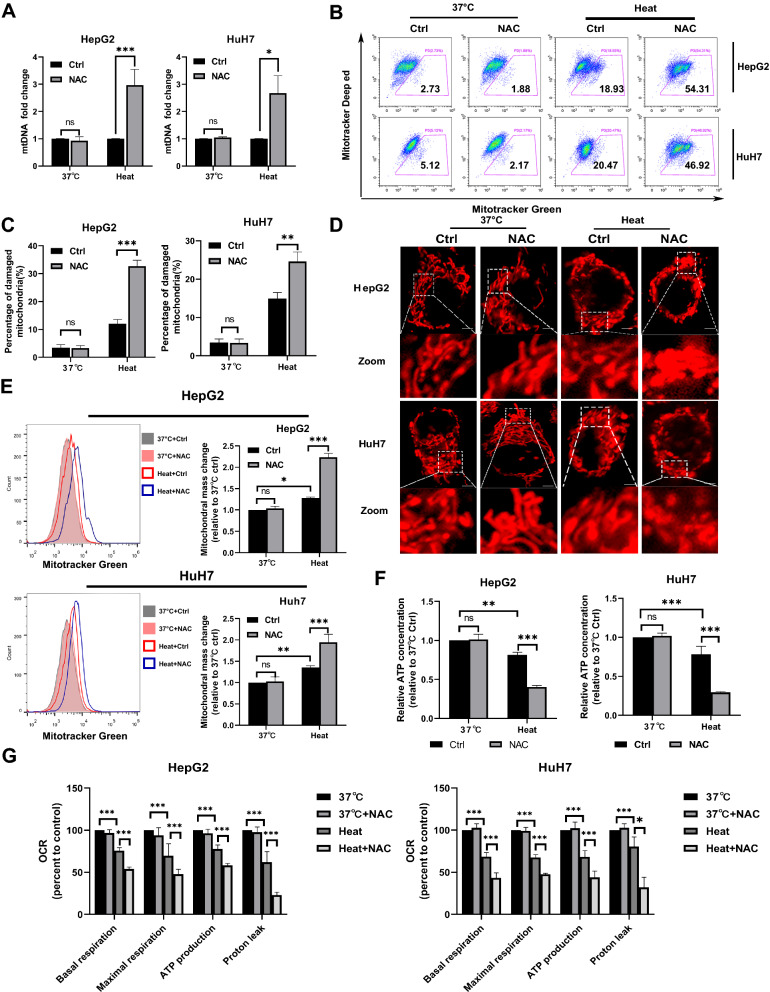
Inhibition of ROS production results in accumulation of damaged mitochondria and severe mitochondrial dysfunction after sublethal heat stress. **A** The mitochondrial DNA copies of each group. **B** Flow cytometry of mitochondrial status. Gates represent cells with damaged mitochondria. **C** The statistical results of **B** in three independent experiments. **D** Representative images of mitochondrial morphology. **E** Mitochondrial as mass assessed by staining with MitoTracker™ Green using a flow cytometric. **F** ATP contents of each group. **G** Results of the Mito stress test. Values are the mean ± SE.*p < 0.05;**p < 0.01; ***p < 0.001; *ns* no significance

Short-term heat exposure can cause damage to the mitochondria [26]. Removal of damaged mitochondria by autophagy is an essential survival response. ROS are known to trigger both autophagy and, mitophay and may be essential for removing damaged mitochondria after sublethal heat stress. We first assessed the content of mtDNA,a biochemical marker of mitochondrial number that is affected by inhibition of ROS after sublethal heat stress. The results showed that NAC treatment after sublethal heat stress increased the mtDNA copy number (Fig. 5A). Then, we further investigated the effect of inhibiting ROS production on removing damaged mitochondria using a fluorescence-based assay. As shown in Fig. 5B, the accumulation of damaged mitochondria was observed in the NAC treatment group after sublethal heat stress(Fig. 5B, C). Then, the morphology, quantity, and function of mitochondria were assessed. As shown in Fig. 5D, the NAC treatment group showed more round mitochondrial structures. Increased mitochondrial mass and reduced ATP production were also observed in the NAC treatment group (Fig. 5E, F). Basel respiration, max respiration, ATP-coupled oxygen consumption and proton leak were significantly decreased in cells by NAC treatment after sublethal heat stress(Fig. 5G). These results suggest that the ROS burst after sublethal heat stress is necessary to remove damaged mitochondria and sustain mitochondrial function.

### Mitophagy is induced by sublethal heat stress and is involved in survival

**Fig. 6:**
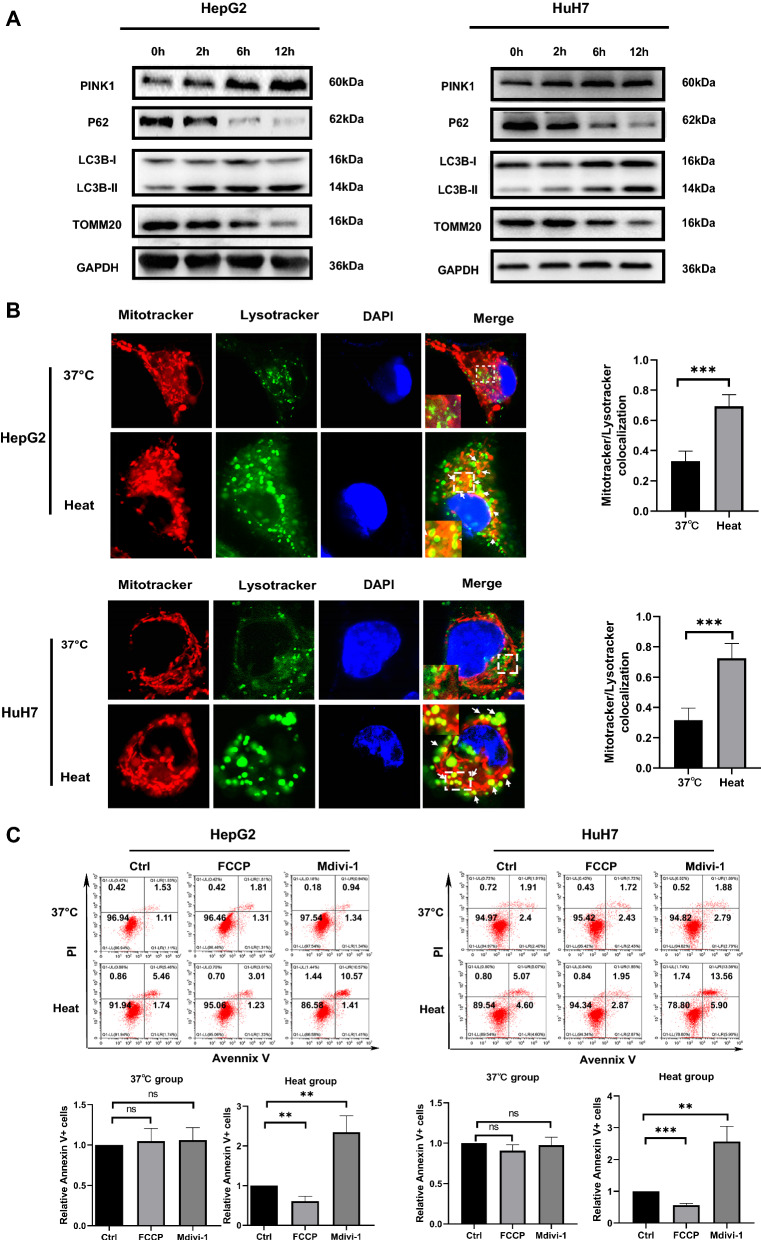
Mitophagy is induced by sublethal heat stress and is involved in survival. **A** WB of GAPDH, P62, LC3B, and TOMM20. **B** Representative confocal images of cells coloaded with MitoTracker™ Red and LysoTracker™ Green at 24 h after sublethal heat stress. Colocalization is shown in yellow and indicated by arrows. **C** Flow cytometry analysis of apoptosis of heated or unheated HCC cells after inducing or inhibiting mitophagy at 24 h after sublethal heat stress. Values are the mean ± SE.**p < 0.01; ***p < 0.001; *ns* no significance

Mitophagy is a type of selective autophagy that mediates the clearance of damaged or dysfunctional mitochondria from the cell. ROS can induce the induction of PINK1-dependent mitophagy. It is unclear whether mitophagy plays a role in cell survival during heat stress. To determine whether mitophagy can be induced by sublethal heat stress, we assessed the LC3B, PINK1, P62, and TOMM20 protein levels via Western blotting. As shown in Fig. 6A, cells exposed to sublethal heat stress showed significantly increased protein expression levels of LC3B-II and PINK1, accompanied by significantly reduced P62 and TOMM20 protein levels. To further verify the induction of mitophagy by sublethal heat stress, we assessed mitophagy after sublethal heat stress based on the colocalization of labeled mitochondria and lysosomes. The results showed that sublethal heat stress exposure caused significant increase in the colocalization of mitochondria and lysosomes (Fig. 6B). This suggests that mitophagy was activated by sublethal heat stress in cells. Therefore, we further investigated whether mitophagy is involved in cell survival after sublethal heat stress. We pretreated cells with FCCP, an inducer of mitophagy, and Mdivi-1,an inhibitor of mitophagy, to observe the changes in apoptosis after sublethal heat stress. As shown in Fig. 6C, inhibition of mitophagy resulted in a 1.5-to 2.5-fold increase in Annexin V + staining, and inducing mitophagy before sublethal heat stress significantly inhibited apoptosis. This confirmed that mitophagy exerts a pro-survival effect after sublethal heat stress.

### Inhibition of ROS production induces apoptosis after sublethal heat stress by inhibiting mitophagy

**Fig. 7:**
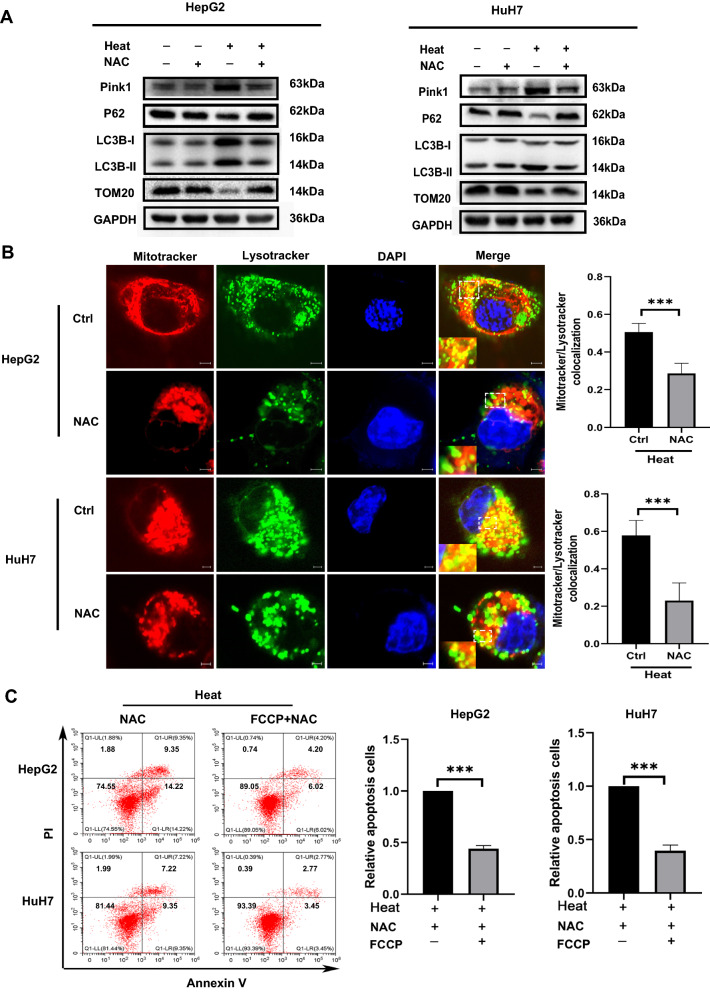
Inhibition of ROS production induces apoptosis after iRFA via mitophagy inhibition. **A** WB of GAPDH, P62, LC3B, and TOMM20 expression in heated or unheated HCC cells treated with or without 5 mM NAC at 24 h after sublethal heat stress (**B**) Representative confocal images of cells coloaded with MitoTracker™ Red and LysoTracker™ green after ROS inhibition. Colocalization is shown in yellow and indicated by arrows. **C** Apoptosis was quantified using Annexin V-FITC/PI staining and flow cytometry of heated cells treated with NAC (5 mM) or FCCP (1 μM) at 24 h after sublethal heat stress. Values are the mean ± SE. ***p < 0.001; *ns* no significance

To determine whether the apoptosis induced by ROS inhibition was mediated by mitophagy, we examined alterations in LC3B-II, PINK1, P62, and TOMM20 protein levels in cells following inhibition of ROS production after sublethal heat stress. As shown in Fig. 7A, decreased PINK1 and LC3B-II protein expression, and P62 and TOMM20 accumulation were observed upon inhibition of ROS production after sublethal heat stress. Moreover, the colocalization between mitochondria and lysosomes was also markedly decreased in the group treated with NAC (Fig. 7B). To further confirm, we examined the impact of the preinduced of mitophagy on NAC induced apoptosis after sublethal heat stress. As shown in Fig. 7C, the effect of NAC on inducing apoptosis after sublethal heat stress was completely eliminated by the predinduction of mitophagy. These data confirmed that mitophagy was suppressed by inhibition of ROS production after sublethal heat stress.

### Nrf2 was elevated in response to oxidative stress after sublethal heat stress and mediated ROS induced mitophagy

**Fig. 8:**
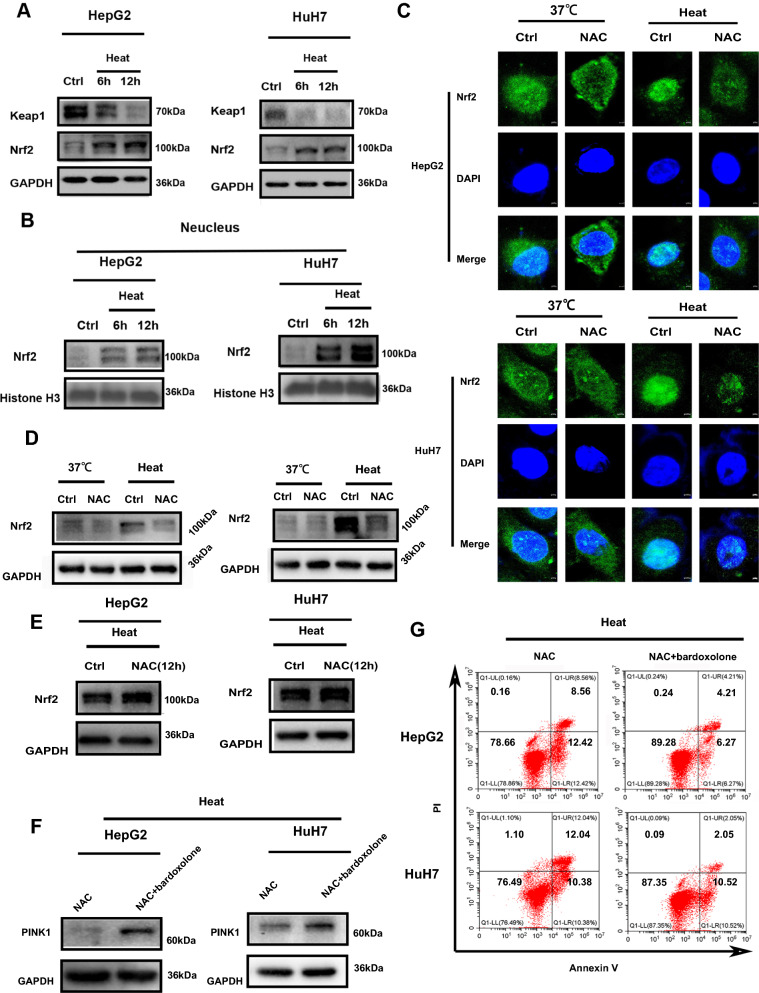
Nrf2 was elevated in response to oxidative stress after sublethal heat stress and mediated ROS induced mitophagy. **A** Western blot of Keap1 and Nrf2 expression in whole cell lysates. **B** Changes in the protein expression of Nrf2 in nucleus. **C** Representative image of Nrf2 localization in cells in different groups.**D**Effect of NAC(5 mM) on Nrf2 **E** Effect of adding NAC(5 mM) 12 h after sublethal heat stress on Nrf2. **F** Inhibition effect of NAC on PINK1 after sublethal heat stress was reversed by bardoxolone. **G** Apoptosis induced by NAC after sublethal heat stress was reversed by bardoxolone at 24 h after sublethal heat stress

Nrf2 is usually activated to promote tumor cell survival under oxidative stress conditions[42].To determine whether the oxidative stress induced by acute sublethal heat stress is sufficient to activate Nrf2, we measured the change in Nrf2 by Western blotting. As shown in Fig. 8A, elevated Nrf2 protein expression was observed after sublethal heat stress and was accompanied by decreased Keap1 expression. Nrf2 is a transcription factor that performs its function in the nucleus. Thus, we futher measured the nuclear protein expression of Nrf2. Increased protein expression of Nrf2 was also observed in the nucleus after sublethal heat stress(Fig. 8B). This was also confirmed by immunofluorescence (Fig. 8C). To determine whether this elevated Nrf2 expression was a responsed to the ROS burst that occurs after sublethal heat stress, we examined the effect of NAC on the protein expression of Nrf2. As shown in Fig. 8C, D, the expression of Nrf2 was significantly inhibited by NAC treatment after sublethal heat stress. We further measured the effect of adding NAC 12 h after sublethal heat stress on the protein expression of Nrf2, since the oxidative stress was confined to within 12 h after sublethal heat stress. The results showed that there was no effect on the protein expression of Nrf2 when NAC was added at 12 h after sublethal heat stress(Fig. 8E). This confirmed that the Nrf2 elevation occurred in response to the ROS burst that occurs after sublethal heat stress. Reports also suggest that Nrf2 can induce PINK1 expression during stress. Thus, we further explored the effect of Nrf2 on the protein expression of PINK1. As shown in Fig. 8F, the NAC-mediated inhibition of PINK1 expression after sublethal heat stress could be reversed by bardoxolone, an activator of Nrf2 (Fig. 8F). Similarly, the apoptosis induced by NAC after sublethal heat stress was also reversed by bardoxolone(Fig. 8G). Taken together, under sublethal heat stress, Nrf2 expression was increased in response to the ROS burst, and then it translocated to the nucleus to induce the expression of PINK1, which is involved in to involve the mitophagy after sublethal heat stress.

### *Inhibition of ROS production sensitizes tumors to iRFA *in vivo

**Fig. 9:**
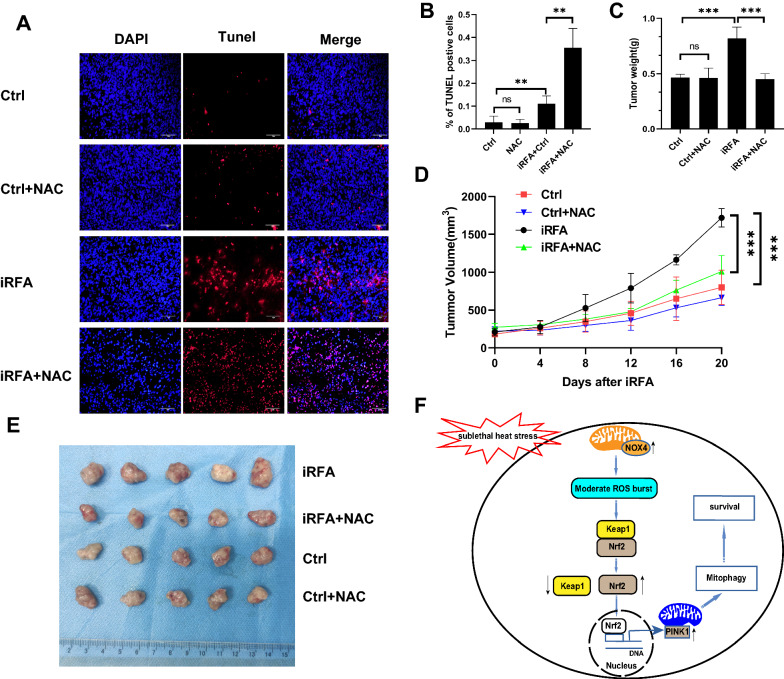
Inhibition of ROS production sensitizes HCC cells to hyperthermia in vivo. NAC (100 mg/kg/day) was administered via tail vein injection starting 2 h after iRFA for 5 days. **A** and **B** A significantly higher portion of TUNEL positive cells was observed in iRFA + NAC Group 1 day after iRFA. The tumors were collected on Day 21. Tumor growth was monitored at the indicated times. The **C** tumor weights and **D** tumor volume growth curves indicated that NAC sensitized HepG2 cells to hyperthermia. **E** Image showing the tumor burdens in mice. **F** Schematic diagram of the ROS burst that is involved in cell survival after sublethal heat stress. Values are the mean ± SE.**p < 0.01;***p < 0.001; *ns* no significance

We then evaluated the effect of inhibition of ROS production on tumor growth in vivo in response to iRFA. Tumors grown from subcutaneously injected HepG2 cells were subjected to iRFA or sham operation. As shown in Fig. 9A, B, administration of NAC after iRFA significantly increased the percentage of TUNEL positive cells. This demonstrated the pro-survival effect of the ROS burst after sublethal heat stress, which was also showed after iRFA in vivo.We then harvested the tumors on Day 21. As shown in Fig. 9C–E, iRFA significantly increased the tumor weight and volume compared to the control group. In the iRFA group, tumor growth was markedly suppressed by the administration of NAC 2 h after treatment. These results show that inhibition of ROS production also sensitizes cells to hyperthermia treatment in vivo.

## Discussion

This study revealed that short-term (within 12 h) elevation in ROS levels exerts a pro-survival effect in HCC after iRFA. The NOX4 isoform of NADPH oxidase located in the mitochondria is responsible for the stimulation of mtROS production by heat stress in HCC cells. Our study also showed that the ROS burst played a pivotal role in the initial induction of PINK1-dependent mitophagy by increasing the expression of Nrf2, and inhibiting the increase in ROS levels after iRFA resulted in the accumulation of damaged mitochondria, which eventually initiated apoptosis.

It is well established that heat stress results in increased ROS levels, which are considered to be important for the induction of cell death [6]. However, the key factor determining whether an adverse effect is induced by stress is the dose–response relationship. The cell-killing effect of hyperthermal treatment is time and temperature dependent [43]. With sublethal heat stress, irreversible cell damage occurs only after prolonged exposure (30 to 60 min) [5]. Clearly, this is not representative of RFA treatment. The thermal dose during iRFA is not sufficient to induce apoptosis. However, increased ROS generation has been observed in cells exposed to sublethal temperatures ranging from 40 to 47 °C regardless of the duration [12, 21, 44–46]. Our data showed that cells suffered short-term oxidative stress after iRFA without cell death. This confirmed that the increased ROS production after iRFA was moderate and noncytotoxic. The apoptosis induced by ROS production inhibition after iRFA demonstrated a pro-survival effect of ROS and confirmed the function of ROS as signaling molecules. This finding is also consistent with previous reports that increased ROS levels can activate prosurvival signaling pathways [10, 16]. Moreover, it has previously been reported that ROS generation at mild temperatures is involved in the induction of cellular defense molecules [12, 47] such as HSPs [18, 21]. Thus, considering that the ROS level after 12 h was still higher than that at pre-iRFA, the ROS burst after iRFA may also protect cells from a higher ROS level post-iRFA by enhancing the antioxidant system.

In our study, we also investigated the source of ROS overproduction in HCC cells after iRFA. These results indicated that ROS were generated by both mitochondria and NADPH oxidases. However, the effects of both DPI and MitoTEMPO on ROS production did not differ from those of NAC, a nonspecific ROS scavenger. This suggests that an upstream and downstream relationship may exist between these two sources of ROS. Further inhibition of NADPH oxidase activity by DPI or VAS2870 attenuated mtROS production, demonstrating an upstream role of NADPH oxidase in mtROS activation. Recent studies have also reported crosstalk between these two producers [39, 48–50]. Indeed, a previous study showed that the mitochondrial inner membrane was still intact, and there was no significant change in the composition of electron transport chain complexes in isolated mitochondria after acute heat treatment [26]. This suggests that the integrity of the ETC in isolated mitochondria may not be affected by short-term heat stress, and there is an factor that initiates increased mtROS production. Recently, “ROS-induced ROS release” was described as a mechanism underlying ROS augmentation [27]. In the present study, we found that NOX4 was upregulated by iRFA, and depletion of NOX4 inhibited iRFA-induced increases in mtROS and total ROS. NOX4 was recently reported to be present in the mitochondria of many types of cells [28, 29, 51]. Our study showed that NOX4 was also located in the mitochondria of HCC cells. This suggests that NOX4 may impact mtROS through ROS generation confined to the mitochondria. Moreover, NOX4 within the mitochondria is regulated by ATP, as described in a previous study [41]. Mitochondrial damage is accompanied by low ATP levels. This indicates that individual generation of ROS in the mitochondria may be highly dependent on ATP and NOX4 levels. Thus, the levels of ROS not only removed the damaged mitochondria, but also avoided the induction of apoptosis.

One of the consequences of increased ROS production is the induction of autophagy [52, 53]. Heat stress has been reported to initiate autophagy in many cell types [54, 55]. Moreover, autophagy has been reported to be a pro-survival mechanism during stress, including iRFA-induced stress [54]. Mitophagy is a type of selective autophagy that mediates the clearance of damaged mitochondria. An increase in ROS levels triggers mitophagy to limit ROS production [56], and we also observed a decline in ROS levels 6 h after iRFA. This may be due to enhanced mitophagy. The mitochondrial outer membrane has been reported to be partially damaged under heat stress conditions (incubation of isolated mitochondria for 20 min at 42 °C) [26]. Our study also showed that mitochondrial damage occurred after heat treatment, and removal of damaged mitochondria by mitophagy is essential for HCC cell survival after iRFA. Consistent with this finding, in *Caenorhabditis elegans* larvae, rebuilding of the mitochondrial network after heat stress has been reported to depend on mitophagy, which is essential for recovery [57]. Moderate mitochondrial ROS levels have been reported to trigger PINK1-dependent mitophagy [23, 24, 58]. In our study, we also observed that inhibition of ROS production blocked PINK1-dependent mitophagy, resulting in accumulation of damaged mitochondria and ultimately inducing apoptosis after iRFA.

Nrf2 plays a vital role in cells encountering oxidative stress, and the protumor role of Nrf2 is also well established [42]. Cells usually adapt acute oxidative stress usually through metabolic reprogramming. Chronic oxidative stress usually activates genetic programs [10].Nrf2 was reported to be activated under persistent moderate heat stress [12]. In our study, Nrf2 was also activated even by acute oxidative stress. Moreover, we also showed a synchronized changes between Nrf2 and PINK1. Over 200 genes can be modulated by Nrf2, including PINK1, whose transcription is upregulated under stress [59, 60].NRF2 has recently been considered to be a key transcription factor of metabolic reprogramming in cancer cells. Activation of NRF2 increases glucose uptake and enhances glycolysis, which is important for the thermotolerance of cells [61]. In addition, Nrf2 affects glutathione synthesis and lipoxidation [62]. Tumors driven by oncogenic KRAS sustain their redox balance through Nrf2 associated glutaminolysis [63, 64]. Moreover, activated Nrf2 increases GPX4, a well established downstream target, and can decrease lipoxidation [65]. Although the role of ferroptosis in iRFA is still unclear, resistance to the ferroptosis inducer sorafenib after iRFA has been reported [66, 67]. This means that increased Nrf2 could also affect ferroptosis. However, this may need to be further explored.

## Conclusions

In summary, we obtained the evidence that increased NOX4 after iRFA significantly increased mtROS production and activated Nrf2 to increase the expression of PINK1, resulting in the induction of mitophagy (Fig. 9F). Enhanced mitophagy removed damaged mitochondria, resulting in increased cell survival after iRFA.

## Data Availability

The original contributions presented in the study are included in the article materials. Further inquiries can be directed to the corresponding authors.

## Abbreviations

HCC: Hepatocellular carcinoma
RFA: Radiofrequency ablation
ROS: Reactive oxygen species
NAC: n-acetylcysteine
DPI: Diphenyleneiodonium chloride
DCFH-DA: 2′,7′-Dichlorodihydrofluorescein diacetate
FCCP: Carbonyl cyanide 4-(trifluoromethoxy) phenylhydrazon
DMSO: Dimethyl sulfoxide
MMP: Mitochondrial membrane potential
GSH: Glutathione
siRNA: Small interfering RNA
iRFA: Insufficient radiofrequency ablation
OCR: Oxygen consumption rate
